# Genetic linkage map construction and QTL identification of juvenile growth traits in *Torreya grandis*

**DOI:** 10.1186/1471-2156-15-S1-S2

**Published:** 2014-06-20

**Authors:** Yanru Zeng, Shengyue Ye, Weiwu Yu, Song Wu, Wei Hou, Rongling Wu, Wensheng Dai, Jun Chang

**Affiliations:** 1 The Nurturing Station for the State Key Laboratory of Subtropical Silviculture, Zhejiang Agricultural and Forestry University, Lin'an, Zhejiang 311300, China; 2 Department of Applied Mathematics Statistics, State University of New York at Stony Brook, Stony Brook, NY 11794, USA; 3 Department of Preventive Medicine, State University of New York at Stony Brook, Stony Brook, NY 11794, USA; 4 Center for Statistical Genetics, Pennsylvania State University, Hershey, PA 17033, USA; 5 Research Institute of Subtropical Forestry, Chinese Academy of Forestry, Fuyang, Zhejiang 311400, China

**Keywords:** Genetic linkage map, Quantitative trait locus, functional mapping, juvenile growth trait, *Torreya grandis*

## Abstract

*Torreya grandis *Fort. ex Lindl, a conifer species widely distributed in Southeastern China, is of high economic value by producing edible, nutrient seeds. However, knowledge about the genome structure and organization of this species is poorly understood, thereby limiting the effective use of its gene resources. Here, we report on a first genetic linkage map for *Torreya grandis *using 96 progeny randomly chosen from a half-sib family of a commercially cultivated variety of this species, *Torreya grandis *Fort. ex Lindl cv. *Merrillii*. The map contains 262 molecular markers, i.e., 75 random amplified polymorphic DNAs (RAPD), 119 inter-simple sequence repeats (ISSR) and 62 amplified fragments length polymorphisms (AFLP), and spans a total of 7,139.9 cM, separated by 10 linkage groups. The linkage map was used to map quantitative trait loci (QTLs) associated with juvenile growth traits by functional mapping. We identified four basal diameter-related QTLs on linkage groups 1, 5 and 9; four height-related QTLs on linkage groups 1, 2, 5 and 8. It was observed that the genetic effects of QTLs on growth traits vary with age, suggesting the dynamic behavior of growth QTLs. Part of the QTLs was found to display a pleiotropic effect on basal diameter growth and height growth.

## Introduction

Plant breeding has proven to be difficult because most economically important traits are quantitatively inherited, controlled by an interacting network of genes, each with a small effect, and environmental factors. This is especially true for forest trees, a group of species characterized by long life spans, high heterozygosity, and environmental heterogeneity [1,2]. It is ubiquitous that variation in phenotypic traits of forest trees fluctuates from year to year owing to varying contributions from genetic and environmental factors. Molecular technologies extensively developed in the past several decades have provided a powerful tool to dissert phenotypic variation into its underlying genetic factors, known as quantitative trait loci (QTLs) [3-5]. Genetic mapping of QTLs from linkage maps has become a routine avenue to identify and study the genetic architecture of phenotypic traits [6].

There has been a considerable body of literature on the construction of genetic maps in forest trees, such as *Eucalyptus *[7], *Salix *[8], *Betula *[9], *Populus *[10-12], and *Pinus *[13,14]. Because it is difficult or impossible to obtain segregating populations from inbred lines, genetic mapping in forest trees should use strategies that are different from those used in annual crops or model systems. These strategies include (1) controlled crosses made from two heterozygous parents, in which different types of markers are segregating [15-17], and (2) open-pollinated progeny from a heterozygous tree, consisted of seeds that share the same mother but have different fathers [14,18]. In gymnosperms including conifers, cycads, ginkgo, and Gnetales, a seed consists of a diploid seed coat from the original parent sporophyte, a haploid megagametophyte developed from a megaspore produced by the original sporophyte, and a diploid embryo developed from the fertilization of the original sporophyte and a male sporophyte [19]. Given its haploid feature, less expensive dominant markers genotyped on the megagametophyte are equally informative to more expensive codominant markers and, therefore, the megagametophyte has been widely used as a tissue to generate molecular markers for genetic mapping.

By taking advantage of megagametophyte-based genotyping, we constructed a low-density genetic linkage map for an important but understudied conifer species, *Torreya grandis *Fort. ex Lindl, using dominant markers. The genus *Torreya *comprises 8 dioecious species, 5 of which originate in China [20]. *T. grandis *cv. *Merrillii *is the only commercially important ever-green variety for nut production. Despite its widespread cultivation in Southeastern China for thousands of years, however, the genetic background of this variety has been poorly understood. In *T. grandis *and *T. grandis *cv. *Merrillii*, only several protocols have been established for molecular marker assays [21-24]. A limited set of markers has been used to estimate genetic relatedness among several varieties [25] and identify sexes in young seedlings [26,27].

The genetic map reported in this study is a first one for *Torreya grandis*. This map was constructed from an open-pollinated progeny, composed of half-sibs, produced from *Torreya grandis *Fort. ex Lindl cv. *Merrillii*. While markers were genotyped from haploid megagametophyte, phenotypic traits were measured from the seedlings of these half-sibs generated from the diploid embryos. Wu [18] derived a statistical model for mapping QTLs for diploid issues using megagametophyte-based markers. We used this model to map QTLs that control stem height and base diameter growth in juvenile seedlings. The dynamic pattern of QTL effects detected was studied by functional mapping, a statistical model for QTL mapping of developmental trajectories [28-32]. The identification of growth QTLs may provide scientific guidance for marker-assisted selection of economically important traits in *Torreya grandis*.

## Results

### Map construction

The 16 primers screened from five random samples generated a total of 109 markers, 91 of which (82%) were polymorphic. The number of polymorphic RAPD markers produced per primer ranged from 2 to 11, with an average of 5.7. ISSR analysis using 24 primers created 194 polymorphic loci in total on the genomic DNAs, with an average of 8 loci per primer. Fifteen polymorphic loci were amplified using primer ISSR-46, but only two using ISSR-52. For AFLP analysis, 14 pairs of selective primers generated 155 polymorphic loci, with E6/M4 amplifying 24 polymorphic loci while E2/M7 and E4/M5 amplifying three only, and an average of polymorphic loci produced by a pair of primers is 11. In total, three different types of markers generated 440 polymorphic loci.

**Figure 1:**
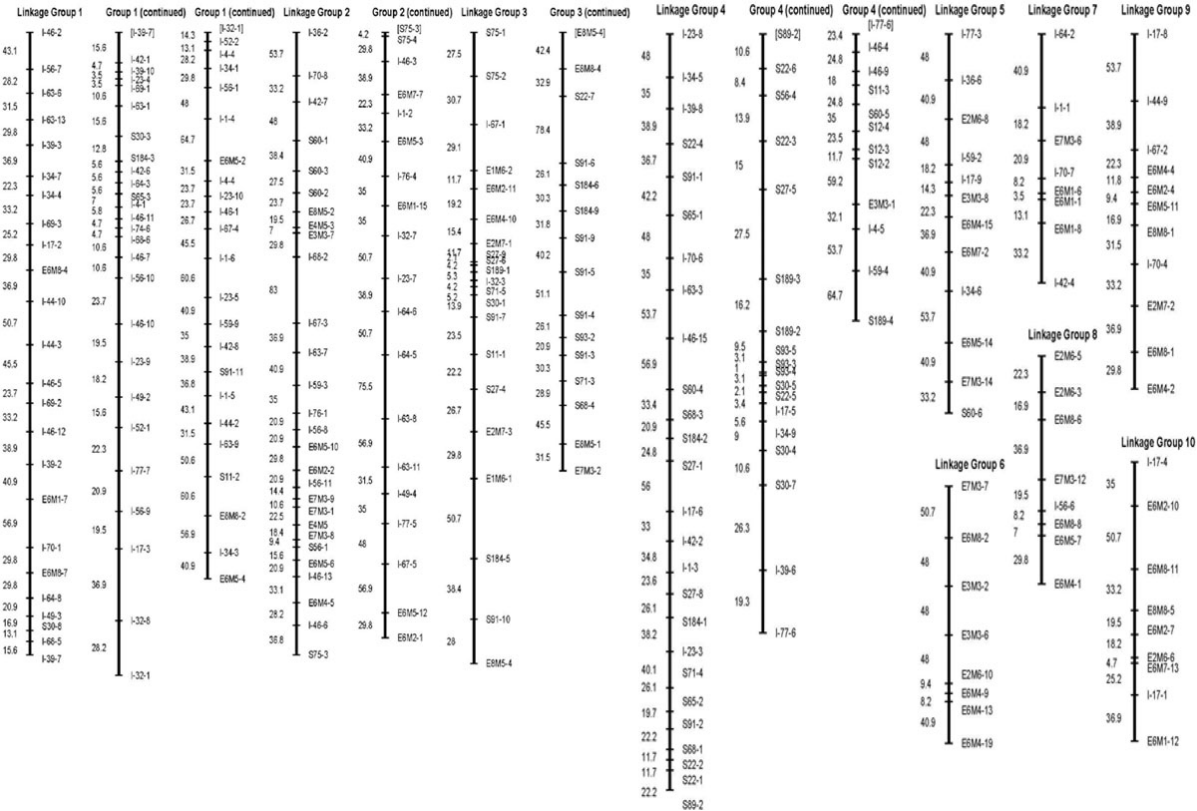
Genetic linkage map constructed from 75 RAPD, 119 ISSR and 68 AFLP markers for 96 half-sib seedlings derived from a maternal parent in *Torreya grandis *cv. *Merrillii*.

Of all the markers, 262 (75 RAPDs, 119 ISSRs and 68 AFLPs) (59%) were identified to follow Mendelian segregation ratio according to chi-square tests at a level of *≥ *= 0.05. These markers were mapped to 10 linkage groups at an LOD > 6 with Mapmaker 3.0. The total map distance was 7,138 cM (Kosambi units) with an average distance between adjacent markers of 28.2cM (Figure 1). Linkage groups each comprise 7 - 71 loci (138 - 1898.5cM). Table 1 shows the distribution of markers in different linkage groups. Our long map constructed in *Torreya grandis *cv. *Merrillii *confirms the detection of large genomes of coniferous species.

**Table 1:** Distribution of molecular markers on 10 linkage groups for a half-sib family of *Torreya grandis *cv. *Merrillii*.

Linkage Group	No. Markers	RAPD	ISSR	AFLP	Max. Distance	Min. Distance	Length	Ave. Distance
LG1	71	6	59	6	64.7	3.5	1898.5	27.1
LG2	46	6	24	16	75.5	4.2	1492.2	33.2
LG3	35	23	2	10	78.4	2.1	915.9	26.9
LG4	54	35	18	1	64.7	1	1394.4	26.4
LG5	12	1	5	6	53.7	14.3	397.3	36.1
LG6	8	0	0	8	50.7	8.2	253.2	36.2
LG7	8	4	4	0	40.9	3.5	138	19.7
LG8	8	0	1	7	36.9	7	140.6	20.1
LG9	11	0	4	7	53.7	9.4	284.4	28.4
LG10	9	0	2	7	50.7	4.7	223.4	27.9

### Detection of growth QTL

**Figure 2:**
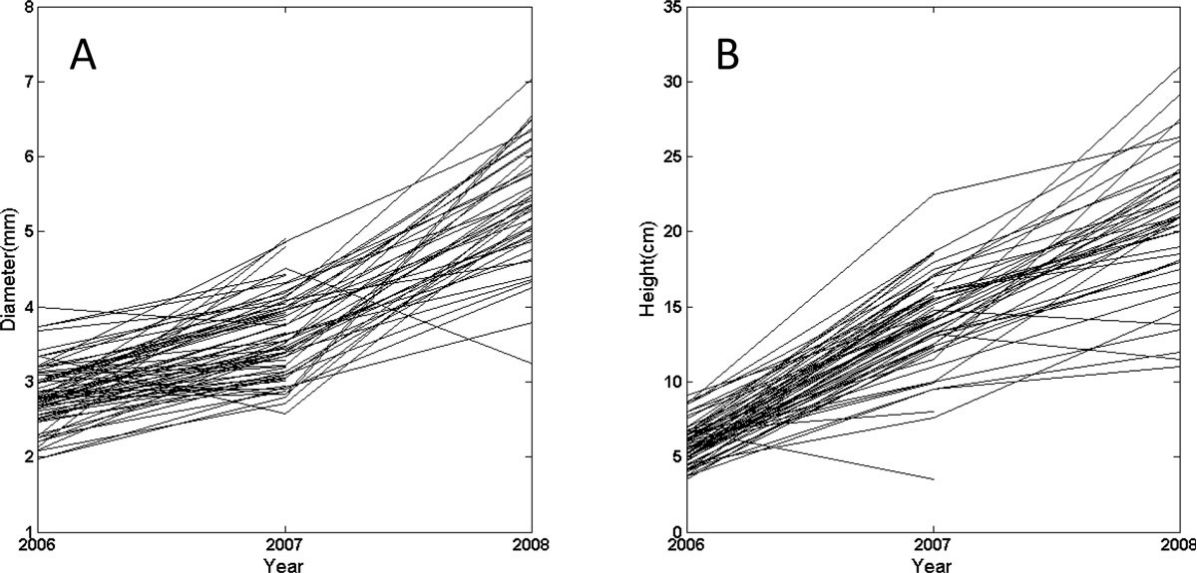
Plots of stem diameter (left) and height growth (right) in the first growing seasons of young seedlings from a half-sib family of *Torreya grandis *cv. *Merrillii*. Part of this material, all with repeated measures at three time points, was used for functional mapping of growth traits.

**Figure 3:**
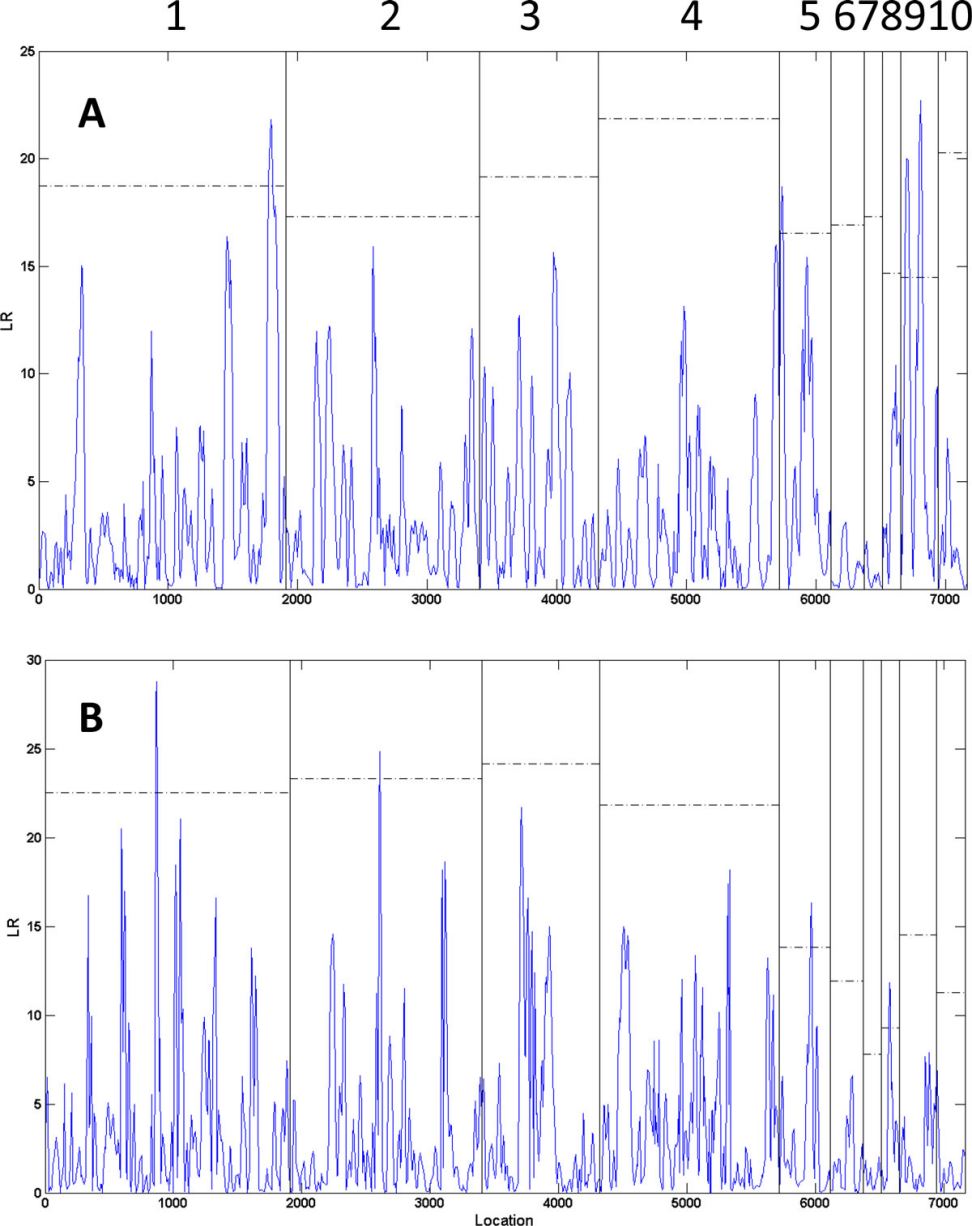
**The profile of log-likelihood ratios (LR) for stem diameter (**A**) and height growth trajectories (**B**) throughout the genome composed of 10 linkage groups in *Torreya grandis *cv. *Merrilli*.** The genomic position corresponding to the peak of the curve is the maximum-likelihood estimate of the QTL location. The 95th percentile (indicated at horizontal lines) of the distribution of the maximum LR values obtained from 200 permutation tests is used as an empirical critical value to declare chromosome-wide existence of a QTL at α = 0.05.

Growth curves of 510 young seedlings from the open-pollinated population over the first three years are illustrated in Figure 2, where substantial variation in curve shape exists, suggesting that specific QTLs may be involved in developmental trajectories. Functional mapping was used to scan the existence and distribution of QTLs for growth traits by calculating genome-wide likelihood ratios (LR) of the full model with a QTL and the reduced model without QTL (Figure 3). By comparing the LR peaks with chromosome-wide critical thresholds determined from permutation tests, we identified several significant QTLs for stem diameter and height growth. As a first QTL study in *T. grandis*, we used a less conservative criterion at the chromosome level to claim the significance of QTLs. Four significant QTLs for stem diameter growth were detected in marker intervals S11-2 - E8M8-2 on linkage group 1, I-77-3 - I-36-6 in linkage group 5, I-17-8 - I-44-9 and E6M5-11 - E8M8-1in linkage group 9. Four significant QTLs for stem height are located between markers I-56-10 - I-46-10 on linkage group 1, I-46-13 - E6M4-5 on linkage group 2, E6M7-2 - I-34-6 on linkage group 5 and E6M8-6 - E7M3-12 on linkage group 8. Linkage group 1 and 5 carry the QTLs for both diameter and height growth, suggesting that these segments may contribute to the genetic correlation between these two growth traits.

**Figure 4:**
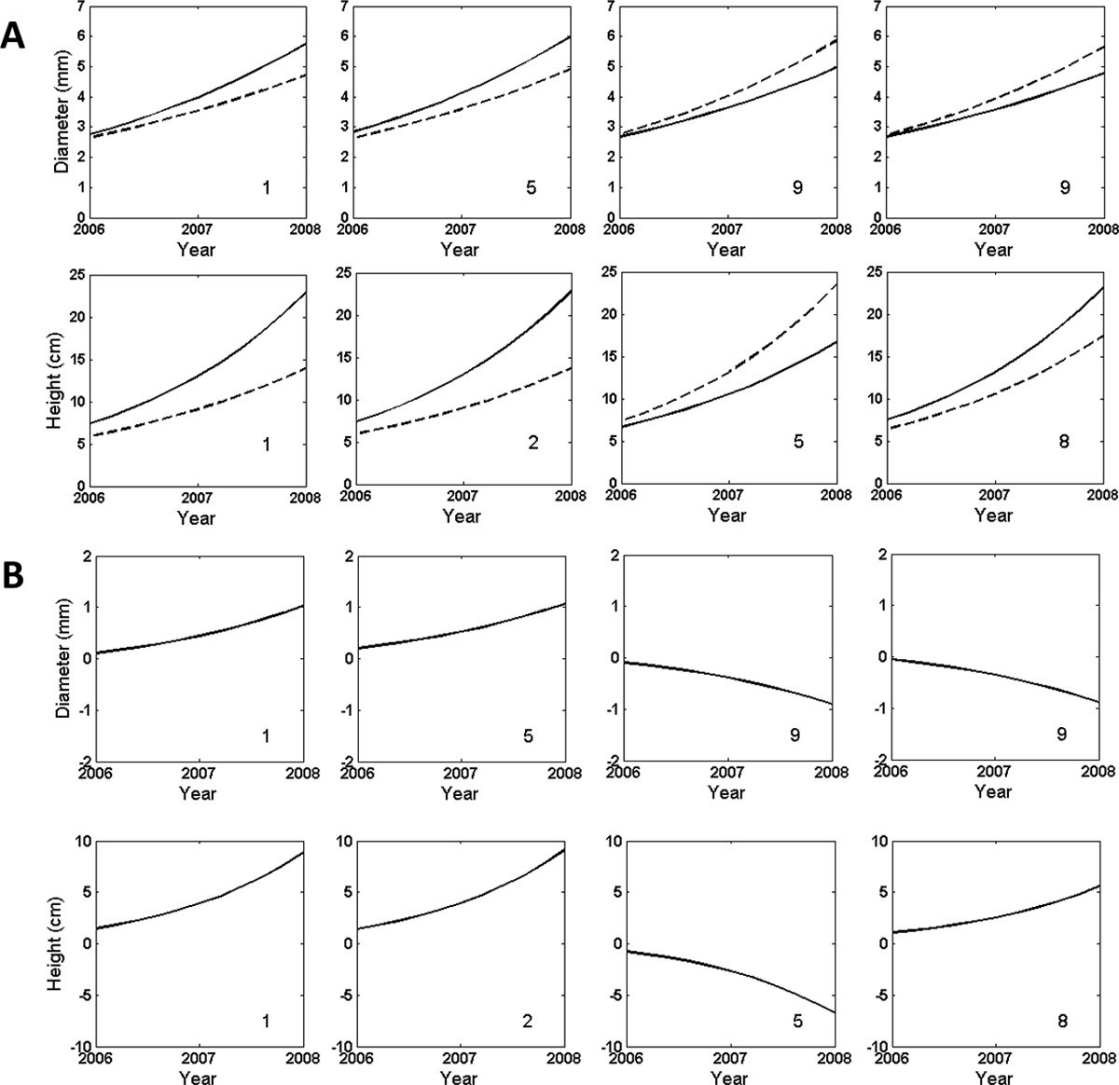
**(**A) **Age-dependent expression profiles of two different genotypes at the QTL detected in different linkage groups for stem diameter and height QTLs in *Torreya grandis *cv. *Merrilli*.** Solid and broken curves represent QTL genotypes *QQ *and *Qq*, respectively. (**B**) Age-dependent genetic effects of the QTLs detected on stem diameter and height growth. The genetic effects are estimated as the differences of genotype *QQ *minus *Qq*.

Using the estimated growth parameters, we drew growth curves of each QTL identified for height and diameter traits (Figure 4A). In general, all genotypes increase in both height and basal diameter with age, although the extent of increase varies depending on the QTL detected. For the height QTLs detected in linkage groups 1, 2, and 8, favorable alleles for increasing stem height are derived from the egg of *T. grandis *cv. *Merrillii*, whereas the favorable allele at the QTL detected in linkage group 5 is from a pollen which may be generated by either *T. grandis *cv. *Merrillii *or another tree in the pool. For the diameter QTLs on linkage groups 1 and 5, the egg of *T. grandis *cv. *Merrillii *contributes favorable alleles for increasing radial growth, whereas two QTLs on linkage group 9 have their favorable alleles from the pollen. As seen in Figure 4B, genetic effects on both diameter and height growth increase with age during the early stage of growth.

## Discussion

Despite its economic, ornamental and ecological importance, the genetic study of *Torreya grandis *has received little attention. For those underrepresented tree species like *T. grandis*, for which little knowledge about their genome structure and sequence is available, simple and cheap dominant markers have still been widely used for constructing genetic linkage maps. In this article, we present a first linkage map for *T. grandis *constructed from three types of dominant markers, RAPD, ISSR and AFLP. A total of 262 markers are clustered into 10 linkage groups, totaling 7,138 cM in length. Since *T. grandis *cv. *Merrillii *has a chromosome number of 2n = 2x = 22 [33], our map only partially covers the genome of *T. grandis*. Also, given its large marker intervals, our map is quite sparse which should be filled by more markers, especially more informative codominant markers. Nevertheless, a long genetic map identified in our study appears to support the big genome of a gymnosperm [34,35]. This map provides an additional fuel to study the structure, organization and evolution of conifer genomes.

We used the linkage map constructed to identify several QTLs for height and radial growth by functional mapping. Although the results presented in this article are preliminary, our study is characterized by two features which are particularly important in genetic mapping of forest trees. First, our mapping design is based on half-sibs from an open-pollinated individual tree in a natural stand. This design can overcome the limitation of genetic mapping for forest trees. Second, while many QTL mapping studies result from the association between markers and phenotypes observed at static points, our method makes use of growth data longitudinally measured at multiple time points through functional mapping [28-32]. Since it was first proposed in 2002, functional mapping has been used to detect growth QTLs in poplar [36] rice [37] and mouse [38]. Its tremendous advantage lies in its integration of biologically meaningful growth equations [39] into QTL mapping, leading to interpretable results of QTL discoveries [40]. All the QTLs identified in this study change their genetic effects over time, with some decreasing while the other increasing.

Powerful genetic and molecular tools have made it possible not only to detect the action of genes at the juvenile stage but also to detect their dynamic actions and interaction of individual loci or QTLs, which is of significance to seedling production in both agriculture and forestry. It has been well known that phenotypic formation results from coordinated action of genes and environmental factors. Based on a better understanding of gene actions, we can design an optimal strategy for seedling selection and production in the first few years in the nursery. Such a strategy is especially important for tree species of highly economic values, like *T. grandis*.

The design used is a half-sib family with members from a heterozygous tree [14,18]. A more powerful design is to sample multiple heterozygous trees randomly from a natural population and further collect open-pollinated seeds from each sampled tree [41]. This design constructs a two-stage hierarchical platform composed of the maternal genotypes and offspring genotypes. Wu et al. [41] implemented a sophisticated procedure of the EM algorithm to provide the simultaneous estimates of linkage disequilibria in natural populations and the QTL-marker linkage based on offspring genotypes. Such a design integrating population genetics and quantitative genetics is especially suitable for genetic mapping in *T. grandis*. More recently, Sun et al. [42] embedded genetic imprinting into this design, enabling geneticists to chart a comprehensive picture of the genetic architecture of growth and morphological traits in forest trees, including *T. grandis*.

## Materials and methods

### Plant material

The mapping population consisted of 510 half-sib progeny of *T. grandis *cv. *Merrillii *from a relatively isolated, open pollinated tree located in Changming Village, Sankou Town, Lin'an City, Zhejiang Province of China. The seeds harvested were piled for 7 days to soften and remove outermost arils and then stratified with sand in an open area to accelerate germination with two layers of plastic film covering on the pile top of the stratified seeds. When the radicle appeared, the seeds were potted in medium (peat: perlite: vermiculitm = 1: 1: 1), followed by partial megagametophyte extraction from germinating seedlings when seedlings were out of top medium. The white megagametophytes extracted were stored at -40°C for DNA isolation later. If megagametophytes are insufficient, fresh young leaves were harvested for DNA isolation. Juvenile growth traits, including seedling height, basal diameter and branching number, were measured consecutively for 3 years at the end of each year.

### Molecular marker analysis

DNA was extracted from both the megagametophytes and young leaves of 96 seedlings by a CTAB method. The quality of DNA extracted was tested by measurement with NanoDrop Sepectrophotomter ND-100 and electrophoresis on 1% agarose gels, based on which the DNA concentration was calculated. The DNAs of the 96 samples were used for RAPD, ISSR and AFLP analyses.

From 100 primers tested in five random samples, a total of 16 RAPD primers were found to produce polymorphic markers. Amplifications were carried out in 20-µl aliquots of the solution containing 12.99 µl H_2_O, 2µl 10×buffer, 1.76 µl 25 m*M *MgCl_2_, 0.3 µl 10 m*M *solution of mixed dNTPs (Sangon Biotech (Shanghai) Co., Ltd.), 0.75µl 10µ*M *primer, 2 µl of a 10 ng/µl DNA solution, and 0.2 µl 5 U/µl *Taq *DNA polymerase (TaKaRa Biotechnology (Dalian) Co., Ltd.). Amplifications were performed in a GeneAmp PCR System 9600 (Applied Biosystem, Singapore) programmed for 40 amplification cycles (94°C for 0.5 min, 35.2°C for 1.5 min, and 72°C for 1.5 min) followed by 7-min extension at 72°C. Amplification products were resolved by electrophoresis on a gel of 1% agarose run at 110 V for 45 min in 1×TBE buffer. Band sizes were estimated by comparison to a GeneRuler 100bp DNA Ladder (Fermentas). Gels were stained with ethidium bromide (EB) and photographed on an Alpha Imager (Alpha Innotech Corporation, USA), and the segregation patterns were scored manually as a band being present or absent from a computer printout. The RAPD markers were denoted by their primer code followed by the numbering of the band (e.g. S30-8).

An AFLP analysis protocol was optimized based on description by Vos *et al*. (1995). A 20 µl aliquot containing 250-ng genomic DNA, 2 µl 10×NE-Buffer 2, 0.2 µl 100×BSA, and 12.35µl H_2_O was digested with the restriction enzymes viz. 0.15µl of a 20 U/µl *Eco*RI (New England Biolabs) and 0.3µl of a 10 U/µl *Mse*I (New England Biolabs) at 37°C for 5 h followed by incubation at 65°C for 15 min to inactivate the enzyme. The DNA digested fragments in the 20 µl aliquot were then ligated at 16°C for 12 hours to 1.4 µl of M-adaptor (50 p*M*) and 1.0 µl of E-adaptor (5 p*M*) contained in a 5 µl aliquot of 0.1µl of a 350 U/µl T4 ligase (TaKaRa Biotechnology (Dalian) Co., Ltd.) and 2.5µl 10×T4 ligase buffer followed by a 10-min enzyme inactivation at 65°C. The pre-selective amplification cycle was carried out in a GeneAmp PCR System 9600 (Applied Biosystem, Singapore) programmed for 25 amplification cycles (94°C for 0.5 min, 56°C for 1 min, and 72°C for 1 min) followed by 5-min extension at 72°C with one selective nucleotide (E-A, M-C) in an 20 µl aliquot containing 2 µl digested fragment solution, 1.5 µl M-primer (10 m*M*), 1.5 µl E-primer (10 m*M*), 2.0 µl 10×PCR buffer, 1.2 µl 25 m*M *MgCl_2_, 0.3 µl 5 U/µl *Taq *polymerase (TaKaRa Biotechnology (Dalian) Co., Ltd.), 1.0 µl 10 m*M *solution of mixed dNTPs (Sangon Biotech (Shanghai) Co., Ltd.), and 10.5µl H_2_O. The pre-selective amplification product was diluted at 1:30 to be used as a template in the following selective amplification. The selective amplification was programmed for initially denaturing at 94°C for 3 min., 15 amplification cycles at 94°C for 0.5 min, 67°C for 0.5 min, and 72°C for 1 min with a lowered annealing temperature of 0.7 each cycle, and 23 cycles at 94°C for 0.5 min, 56°C for 0.5 min, and 72°C for 1 min followed by 7-min extension at 72°C using a pair of primers, each of which contained three selective nucleotides, in a 20 µl aliquot containing 2 µl diluted pre-amplification product, 1.4 µl M-primer (10 m*M*), 1 µl E-primer (10 m*M*), 2.0 µl 10×PCR buffer, 1.3 µl 25 m*M *MgCl_2_, 0.2 µl 5 U/µl *Taq *polymerase (TaKaRa Biotechnology (Dalian) Co., Ltd.), 1.0 µl 10 m*M *solution of mixed dNTPs (Sangon Biotech (Shanghai) Co., Ltd.), and 11.1µl H_2_O. Totally 41 pairs of primers were used in the experiment. The amplification products thus obtained were run on a 6% polyacrylamide gel for 2.5 h on a sequencer (CBS, USA). Gel images were scanned and manually scored for the presence or absence of bands. The AFLP bands were named after the code of *Eco*R I/*Mse *I primer pairs followed by the number of the band.

In total, 24 ISSR primers were used in the analysis of genomic DNAs. Amplifications were performed in 20-µl aliquots of the solution, each of which contained 3 µl of a 10 ng/µl template DNA, 2 µl 10×buffer, 3.25 µl 25 m*M *MgCl_2_, 0.5 µl 25 m*M *solution of each dNTP (Sangon Biotech (Shanghai) Co., Ltd.), 0.7µl 10 µ*M *primer, 0.2 µl *Taq *DNA polymerase (TaKaRa Biotechnology (Dalian) Co., Ltd.) and 10.35 µl H_2_O. Amplifications were programmed for pre-denaturing at 94°C for 5 min, and 35 amplification cycles (94°C for 30 s, denaturing for 45 s, and 72°C for 90 s) followed by 7-min extension at 72°C, in which the denaturing temperature varied among primers. Amplification products were resolved by electrophoresis on a gel of 1.6% agarose at 150 V for 30 min, EB stained and photographed (Alpha Innotech Corporation, USA). The segregation patterns of ISSR markers were scored and denoted in a way similar to those of RAPD makers.

### Map construction and QTL mapping

Chi-squared tests were performed to check if the markers genotyped follow the Mendelian ratios. The markers with significant departure from Mendelian segregation ratios were excluded from the map construction. A maternal linkage map was constructed using MapMaker version 3.0 [43] following a backcross mapping strategy. The data sets of dominant markers were duplicated to allow the detection of repulsion phase between linked markers, indicated by *R *after the names of markers. The "triple error detection" and the "error detection" features were used to recognize the circumstance in which an event was more probably the result of error than of recombination. These features avoid map expansion [44]. Linkage groups were assigned with thresholds for a minimum LOD (logarithm of the odds) score of 6.0 and a maximal recombination fraction of 0.30. The markers were ordered using the "Order" command iteratively with a default LOD of 3.0. The first-sequence order was confirmed using the "Ripple" command permuting five markers at a time. All markers that were not ordered in the first pass were placed again using the "Try" command. Linkage maps were generated with the "map" command using the Kosambi mapping function. Maps were drawn with the program MapChart 2.1 [45].

For megagametophyte-generated markers, we used Wu's [18] approach to formulate a mixture likelihood for functional mapping of QTLs that control growth traits. Functional mapping is a dynamic model that was derived to map growth QTLs through integrating growth equations into a mapping framework [28-32]. In this study, growth traits were measured in the first three years of growth. Growth curves that describe juvenile growth are defined by an exponential equation [39], i.e.,

$g\left(t\right)=ae^{rt}$

where *g*(*t*) is the growth of a trait at time *t, a *is the constant and *r *is the exponent. Thus, by estimating *a *and *r *for each QTL genotype, we can test the effect of a QTL on growth curves.

Functional mapping also models the covariance structure of growth traits measured at multiple time points by a statistical approach. In this study, we implemented a first-order autoregressive (AR (1)) model to fit the covariance structure by using the variance and the between-time correlation. To make the data fitted by the stationarity of covariance and correlation assumed for the AR (1) model, we log-transformed raw data of growth. The log-transformation leads to more parallel curves and, thus, increases the feasibility of using the analytically advantageous AR (1) model to analyze the growth data.

If different genotypes at a given QTL correspond to different trajectories, the QTL must affect the differentiation of this trait. Therefore, by estimating the curve parameters (*a, r*) that define the trait trajectory of each QTL genotype and testing the differences in these parameters among genotypes, we can determine whether a dynamic QTL exists and how it affects the formation and expression of a trait during development.

## Competing interests

The authors declared that they have no competing interests.

## Authors' contributions

Conceived and designed the experiments: YZ RW. Performed the experiments: YZ SY WY. Analyzed the data: SW, WH. Contributed reagents/materials/analysis tools: YZ WD JC. Wrote the paper: YZ RW.

## Funding

Publication of this work was supported by grants (2008C22003 and 2012C12904-12) from Department of Science and Technology, Zhejiang Province, China, NSF/IOS-0923975 and "863" Program 2013AA102605 of China.

## Declarations

This article has been published as part of *BMC Genetics *Volume 15 Supplement 1, 2014: Selected articles from the International Symposium on Quantitative Genetics and Genomics of Woody Plants. The full contents of the supplement are available online at http://www.biomedcentral.com/bmcgenet/supplements/15/S1.
